# Association of life course socioeconomic status and adult height with cognitive functioning of older adults in India and China

**DOI:** 10.1186/s12877-021-02303-w

**Published:** 2021-06-09

**Authors:** Y. Selvamani, P. Arokiasamy

**Affiliations:** 1 International Institute for Population Sciences (IIPS), Govandi Station Road, Mumbai, 400088 India; 2 Department of Development Studies, International Institute for Population Sciences (IIPS), Govandi Station Road, Mumbai, 400088 India

**Keywords:** Childhood socioeconomic status, Stature, Cognition, Intrinsic capacity, WHO-SAGE, Ageing

## Abstract

**Background:**

Cognitive functioning is an important measure of intrinsic capacity. In this study, we examine the association of life course socioeconomic status (SES) and height with cognitive functioning among older adults (50+) in India and China. The age pattern of cognitive functioning with measures of life course socioeconomic status has also been examined.

**Methods:**

Cross-sectional comparative analysis was conducted using the WHO’s Study on global AGEing and adult health (SAGE) data for India and China. Multilevel mixed-effect linear regression analysis was used to examine the association of life course socioeconomic status and adult height with cognitive functioning.

**Results:**

In both India and China, parental education as a measure of childhood socioeconomic status was positively associated with cognitive functioning. The association between adult socioeconomic status and cognitive functioning was positive and significant. Height was significantly and positively associated with improved cognitive functioning of older adults in India and China. Furthermore, the age-related decline in cognitive functioning score was higher among older adults whose parents had no schooling, particularly in China. The cognitive functioning score with age was much lower among less-educated older adults than those with higher levels of education in China. Wealthier older adults in India had higher cognitive functioning in middle ages, however, wealth differences narrowed with age.

**Conclusions:**

The results of this study suggest a significant association of lifetime socioeconomic status and cumulative net nutrition on later-life cognitive functioning in middle-income settings.

**Supplementary Information:**

The online version contains supplementary material available at 10.1186/s12877-021-02303-w.

## Background

Rapid demographic changes such as the reduction in fertility and improvements in health have resulted in the rise of life expectancy both in India and China, the two most populated nations in the world with more than a third of the global population. In addition to the one child policy, the rise in life expectancy was faster in China than in India and as a result, the share of the elderly population is rising rapidly [1]. By 2050, the projected elderly population (60+) to reach 36.5% in China and 19.4% in India, respectively [2].

The age-associated changes in mental health conditions such as cognitive impairment, depression and dementia are significant contributors to the global disease burden [3–5]. Individuals who maintain better cognitive functioning in early years and old age have better outcomes such as improved quality of life, lower risk of disabilities and all-cause mortality [6–10]. While cognitive functioning is an important measure of intrinsic capacity [11], evidence suggests that the secular improvement in the levels of cognitive functioning [12] and socioeconomic position across the globe [13, 14]. Individual improvement in cognition is mainly driven by conditions during childhood and adult life, such as improvement in childhood health, nutrition, and better socioeconomic conditions and structural factors [15–19].

A growing body of literature suggests a significant association between childhood socioeconomic status and health conditions with cognitive functioning in later life. Childhood socioeconomic status plays an important role in determining the higher educational attainment, immunisation, health, and nutrition which have a significant impact in later life [20–23]. Studies mainly from high-income countries showed strong association of adverse childhood circumstances such as poor socioeconomic status, and childhood poverty/deprivation with poor cognitive functioning, cognitive impairment, and dementia [24–31]. However, very few studies have examined the association between life course socioeconomic status and cognitive functioning in low and middle-income countries [32].

Furthermore, literature shows the linkages of height with health, physical, and cognitive functioning. Adult height is a summary measure of health and net nutrition in early childhood [33–35]. The height of the individual makes significant difference from childhood on various outcomes of health and wellbeing [36]. Taller children perform well in school, sports, and cognitive functioning tests, and secure higher positions. On average taller people have a more economic advantage than short people; taller people earn more than their shorter counterparts [37, 38]. Also, taller older adults have higher cognitive functioning than their shorter counterparts [39, 40] and are at lower risk of developing dementia [41–43]. It is also notable that the income-height relationship mediates through higher cognitive functioning [37]. Height is also associated with better health, happiness, and overall quality of life [44–46].

Studies reflecting association between the life course socioeconomic status and height and cognitive functioning are limited in middle-income countries. In this paper, we examine the association of childhood socioeconomic status (parental education and employment), adult height, and adult socioeconomic status (own education and wealth quintile) with cognitive functioning among older adults in two middle-income countries, namely, India and China using WHO-SAGE Wave 1 data. We also examine the age pattern of cognitive functioning score across the life course socioeconomic conditions.

## Methods

### Data and sample

#### WHO study on global AGEing and adult health (SAGE)

In this paper, data from the WHO’s SAGE survey, a nationally representative household health survey conducted in six low and middle-income countries: China, Ghana, India, Mexico, the Russian Federation, and South Africa during 2007–10 is used. SAGE data was collected by the World Health Organisation (WHO) with support from national and international organisations. The main aim of SAGE survey was to fulfil the data gaps and understand the health and well-being of the growing ageing population in the six low and middle-income countries. SAGE measures are comparable with other studies from high-income countries such as the Health and Retirement Study (HRS), and the Survey of Health, Ageing and Retirement in Europe (SHARE). A multistage, stratified clustered sample design was used homogeneously in all the countries to collect the data from the older adults. SAGE included a sample of 34,124 older adults aged 50 and above and a comparative sample of 8307 adults aged 18–49.SAGE collected data on self-reported as well as biomarkers on different domains of health, wellbeing, and anthropometric indicators. Height was measured by trained health investigators. Besides, SAGE also collected data on parental characteristics such as parental education and employment. This analysis was conducted on the cross-sectional sample of 19,666 older adults aged 50 years and above for India (*n* = 6560) and China (*n* = 13,106) based on WHO-SAGE Wave 1 data conducted during 2007–10. SAGE is a longitudinal survey, however, at the time of the data analysis, only Wave 1 data was available. More detailed information on sampling, methodology and data are provided in Kowal et al. (2012) [47].

The SAGE study was approved by the Ethics Review Committee (RPC146), World Health Organization, Geneva, Switzerland and the Institutional Review Board, International Institute of Population Sciences, Mumbai, India and the ethics review committee of the Chinese Canter for Disease Control and Prevention (China CDC) (Approval notice 200,601).

### Outcome variable

#### Cognitive functioning

SAGE survey collected information on cognition measures such as immediate and delayed verbal recall, verbal fluency, and forward and backward digit span. In this analysis, we generated a standardised cognitive function index combining the variables covering three domains of cognition using principal components analysis and the final score of this index ranged from 0 to 100; higher scores represent higher cognitive functioning [48]. A detailed description of the process of cognitive functioning variable construction is presented in the supplementary file.

### Predictor variables

#### Childhood socio-economic status (SES)

SAGE collected data on parental education and work status/employer for mother and father. The parental education responses were captured in seven categories from no formal education to post-graduation. For the analysis, we categorized parental education into four categories: no formal education, less than primary, completed primary or secondary, and completed higher secondary (HS) or above. Parental employment was recoded into four categories; not employed, self-employed, employed in the informal sector, employed in the private sector/public sector.

#### Height

In the SAGE survey, height was measured in centimetres using a stadiometer by trained health investigators. In the analysis, sex and country-specific height quintiles were generated to examine the association between height and cognitive functioning in India and China. The height quintile distribution of the study population for India and China is presented in the supplementary file.

#### Measures of adult SES

In this study, educational attainment, wealth quintile and work status have been included as measures of adult socioeconomic status. Educational attainment was categorized as ‘no formal education’, ‘less than primary’, ‘completed primary or secondary’, and ‘completed higher secondary (HS) or above’. Wealth quintile variable was generated from measures of household amenities and ownership of durable goods and categorised as ‘poorest’, ‘poorer’, ‘middle’, ‘richer’, and ‘richest’. A list of household wealth variables was used to calculate the wealth quintile which is provided in supplementary Table 1. The work status of the study participants was categorised as ‘not employed’, ‘self-employed’, ‘informal sector’, and ‘private sector/public sector’.

#### Life course SES

We generated a life course SES variable to understand the social mobility and cognitive functioning among the older adults in India and China [49]. Education-based life course SES was generated by combining parental and own education and defined as: 1. ‘Stable low’ when parental and respondents’ education was less than primary; 2. ‘Declining’ when parental education is greater than primary and respondents’ education was less than primary; 3. ‘Increasing ‘when parental education was less than primary and respondents’ education was greater than primary; 4. ‘Stable high ‘when parental education was greater than primary and respondent’s education was greater than primary. Employment-based life course SES was generated by combining parental and own employment status and defined as: 1. ‘Stable low’ when parent and the respondent were both not employed; 2. ‘Declining’ when a parent was employed and the respondent was not employed; 3. ‘Increasing’ when a parent was not employed and respondent was employed; 4. ‘Stable high’ when parent and respondent were both employed. The distribution of sample according to life course SES variables is presented in supplementary file.

### Demographic and health characteristics

We included selected demographic and health variables as covariates which include age (years), place of residence (urban/rural), marital status (currently married/otherwise), body mass index (underweight (< 18.5 kg/m^2^), normal (18.5–24.9 kg/m^2^), overweight (25.0–29.9 kg/m^2^), and obesity (30.0+ kg/m^2^)), poor self-rated health (SRH) (no/yes). Self-reported depression (no/yes).

#### Activities of daily living (1 + ADL) limitations

Data on the questions measuring difficulties in doing activities in the last 30 days have been used to generate”1 + ADL limitations’ variable. These questions captured difficulties in ‘sitting for long periods’, ‘walking 100 meters’, ‘standing up from sitting down’, ‘standing for long periods’, ‘climbing one flight of stairs without resting’, ‘stooping/kneeling/crouching’, ‘picking up things with fingers’, ‘extending arms above shoulders’, ‘concentrating for 10 min’, ‘walking a long distance (1 km)’, ‘bathing, getting dressed’, ‘carrying things’, ‘moving around inside home’, ‘getting up from lying down’, and ‘getting to and using the toilet’. We recoded severe and extreme difficulties to represent difficulties in activities of daily living. Further, we summed up these measures into one variable and coded “0″ as no difficulty” else (one or more) into “1″ to represent 1+ ADL limitations.

#### Sleep problems

In the SAGE survey, the prevalence of sleep problems was determined with the following question ‘Overall in the last 30 days, how much of a problem did you have with sleeping, such as falling asleep, waking up frequently during the night or waking up too early in the morning?’ response categories were none, mild, moderate, severe, extreme/cannot do. Those who reported ‘severe’ and ‘extreme/cannot do’ were considered as having sleeping problems.

#### Edentulism (loss of all natural teeth)

The prevalence of complete tooth loss was assessed with the following question. ‘Have you lost all of your natural teeth?’ Those who said yes were coded as 1 “edentulous” else into 0 “dentate’.

### Health Behaviours

#### Physically inactive

Those who reported physical activity less than 300 min in a week are considered as physically inactive. ***Tobacco use*** was categorised as yes or no. ***Alcohol consumption*** was categorised as yes if the respondent consumed 1–4 days/week or more in the last 12 months’ or no.

### Statistical analysis

Bivariate analysis was used to understand the sample distribution by background characteristics. Further, we assessed the association of life course socioeconomic status and height (quintile) and cognitive functioning using multilevel mixed-effect regression models. Three-level random intercept regression models were used which included province at the first level, Primary Sampling Unit (PSU) at second, and individual at the third level. Furthermore, to understand the age patterns of cognitive functioning trajectories by life course socioeconomic status, the interaction of life course socioeconomic status measures such as parental education and adult socioeconomic status (schooling and wealth quintile) with age were used. All analyses were carried out in STATA 15.0.

## Results

### Study participants

Descriptive statistics of the study participants are provided in Table 1. The mean age of study participants was 61.5 years in India and 61.6 years in China. Older adults in China were slightly taller than their counterparts in India. The distribution of the sample by place of residence showed that more than half of the sample were from rural areas in India (71%) and China (53%). A large proportion of study participants in China were currently married (85.0%) as compared to 77% in India. In India, more than half of older adults (51%) had no formal education. About 27% of older adults in India have never worked. In India and China, around 90% of the participant’s mother had no schooling. In India and China, more than 65% of the study participant’s father had no schooling. In India, about 65% of the study participant’s mother never worked, compared to 38.7% in China. A higher percentage of older adults in India reported sleep problems (14.5%), compared to 2.7% in China. Similarly, the prevalence rate of loss of all natural teeth (edentulism) was higher in India (15%) and a large proportion of the study participants in India were underweight (38%). The prevalence of poor self-rated health among study participants was 22.4 and 21.2% in India and China, respectively. More than half of the study participants in India reported 1 + ADL limitations. The prevalence of tobacco use was higher in India (47%) and the prevalence of physical inactivity was higher in China.

**Table 1:** **Characteristics of the study population, WHO-SAGE Wave 1 (2007/10)**

Background Characteristics	Categories	India	China
Mean age (years, SD)		61.5 (8.89)	62.6(8.96)
Mean height (cm, SD)		156.7(9.97)	159.2(8.68)
Mean cognitive functioning (score, SD)		38.1 (10.01)	50.9 (11.8)
–		%	%
Sex	Male	51.0	49.8
	Female	49.0	50.2
Residence	Urban	28.9	47.4
	Rural	71.1	52.6
Marital status	Married	76.9	85.1
	Otherwise	23.1	14.9
Schooling	No formal education	50.8	22.6
	Less than primary	10.9	18.8
	Completed secondary	24.4	40.9
	Higher secondary (HS) and above	13.9	17.7
Wealth quintile	Poorest	18.2	16.3
	Poorer	19.5	18.1
	Middle	18.8	20.5
	Richer	19.6	23.3
	Richest	23.9	21.8
Own employment	Never worked	27.0	8.6
	Informal employment	22.2	2.6
	Self-employed	36.4	45.2
	Private/public sector	14.4	43.6
Mother’s education	No formal education	90.2	87.4
	Less than primary	5.3	6.0
	Completed secondary	3.9	5.1
	Higher secondary (HS) and above	0.6	1.5
Father’s education	No formal education	66.6	68.7
	Less than primary	13.3	12.9
	Completed secondary	15.4	13.9
	Higher secondary (HS) and above	4.8	4.5
Mother’s employment	Never worked	65.5	38.7
	Informal employment	17.6	1.8
	Self-employed	14.5	48.6
	Private/public sector	2.5	10.9
Father’s employment	Never worked	2.8	24.8
	Informal employment	26.4	2.0
	Self-employed	57.1	51.1
	Private/public sector	13.7	22.1
Body mass index	Underweight	38.3	4.1
	Normal weight	48.3	59.9
	Overweight	10.6	29.9
	Obesity	2.8	6.1
Self-rated health	Good	77.6	78.8
	Poor	22.4	21.2
1 + ADL	No	47.8	87.1
	Yes	52.2	12.9
Sleep problems	No	85.5	97.3
	Yes	14.5	2.7
Edentulism (Teeth loss)	No	84.9	90.9
	Yes	15.1	9.1
Tobacco use	No	53.1	73.6
	Yes	46.9	26.4
Alcohol use	No	96.0	83.2
	Yes	4.0	16.8
Physical inactivity	No	68.5	61.2
	Yes	31.5	38.8
Self-reported depression	No	95.9	99.7
	Yes	4.1	0.3
Observations	–	6560	13,106

**Fig. 1:**
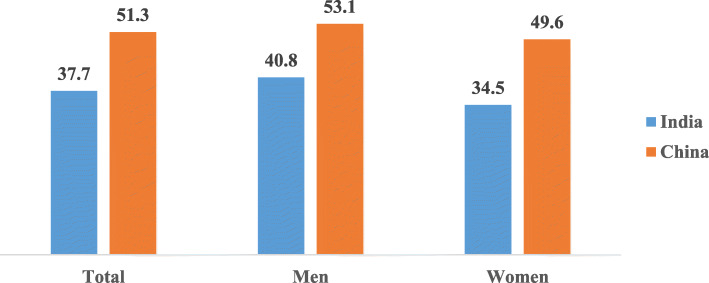
Age-adjusted mean cognitive functioning score among older adults in India and China, WHO-SAGE Wave 1 (2007/10)

The overall age-adjusted mean cognitive functioning score was higher among older adults in China (51.3) than India counterparts (37.7). Older women in India and China had lower cognitive functioning score than men (Fig. 1). The correlation between the main outcome variable (cognitive functioning) and measures of life course socioeconomic status, height, demographic and health measures is presented in supplementary file.

### Results from multivariate analysis

Results from multilevel multivariate regression models showed that parental education was significantly and positively associated with late-life cognitive functioning in India and China **(**Table 2**).** The association between father’s education and cognitive functioning was strong and positive. Older adults in India and China whose fathers completed higher secondary (HS) or above had higher cognitive functioning score (β = 2.53, CI: 1.36, 3.69, *p* < .001) and (β = 3.50, CI: 2.43, 4.57, p < .001) than of older adults whose fathers had no schooling, respectively. Similarly, older adults in India and China whose mothers completed high school or above had 3.07 (CI: 0.59, 5.56, *p* < .005) and 2.17 (CI: 0.54, 3.80, *p* < .001) higher cognitive functioning score than of older adults whose mothers had no schooling.

**Table 2:** Mixed effect linear regression results of cognitive functioning among older adults for India and China, WHO-SAGE Wave 1 (2007/10)

Characteristics	Categories	India	China
–	–	β (95% CI)	β (95% CI)
Mother’s education^a^	Less than primary	0.85*(−0.04, 1.76)	1.50***(0.69, 2.31)
	Completed secondary	1.53***(0.40, 2.66)	0.96**(0.022, 1.90)
	Higher secondary (HS) and above	3.07**(0.59, 5.56)	2.17***(0.54, 3.80)
Father’s education^a^	Less than primary	0.80**(0.18, 1.43)	1.35***(0.76, 1.94)
	Completed secondary	1.00***(0.31, 1.68)	1.24***(0.60, 1.88)
	Higher secondary (HS) and above	2.53***(1.36, 3.69)	3.50***(2.43, 4.57)
Mother’s employment^b^	Informal employment	0.38(−0.38, 1.15)	− 0.016(− 1.95, 1.92)
	Self-employed	− 0.29(− 0.94, 0.36)	0.36(− 0.42, 1.15)
	Private/public sector	− 0.77(−2.11, 0.56)	0.26(− 0.50, 1.02)
Father’s employment^b^	Informal employment	1.31**(0.038, 2.59)	0.61(−1.15, 2.38)
	Self-employed	2.15***(0.93, 3.37)	0.62(−0.17, 1.41)
	Private/public sector	2.04***(0.71, 3.37)	0.40(−0.31, 1.11)
Height quintile^c^	2	0.83**(0.20, 1.46)	0.96***(0.39, 1.52)
	3	1.06***(0.42, 1.70)	1.32***(0.76, 1.89)
	4	1.38***(0.74, 2.02)	1.63***(1.03, 2.23)
	Highest	1.81***(1.15, 2.47)	2.23***(1.60, 2.86)
Schooling^a^	Less than primary	3.80***(3.11, 4.49)	2.96***(2.39, 3.53)
	Completed secondary	5.63***(5.03, 6.23)	5.44***(4.90, 5.99)
	Higher secondary (HS) and above	9.28***(8.45, 10.1)	7.82***(7.08, 8.55)
Wealth quintile^d^	Poorer	0.92***(0.24, 1.61)	0.75***(0.18, 1.33)
	Middle	1.42***(0.71, 2.13)	1.02***(0.40, 1.64)
	Richer	2.01***(1.28, 2.74)	2.28***(1.63, 2.93)
	Richest	2.71***(1.93, 3.49)	2.07***(1.34, 2.81)
Own employment^b^	Informal employment	0.56(−0.16, 1.29)	1.21*(−0.11, 2.54)
	Self-employed	0.42(−0.23, 1.07)	0.39(−0.42, 1.21)
	Private/public sector	1.21***(0.44, 1.98)	2.47***(1.68, 3.25)
Age (years)		−0.08***(− 0.11, − 0.06)	−0.26***(− 0.28, − 0.23)
Gender^e^	Female	−2.96***(−3.54, −2.38)	−1.57***(− 2.02, −1.12)
Residence^f^	Rural	−0.72**(− 1.42, − 0.019)	−0.73(− 2.14, 0.68)
Marital status^g^	Otherwise	−1.09***(− 1.60, − 0.57)	−0.82***(− 1.33, − 0.31)
Body mass index^h^	Underweight	−0.86***(− 1.31, − 0.40)	−0.15(− 1.04, 0.72)
	Overweight	0.89***(0.24, 1.54)	0.33*(−0.06, 0.73)
	Obesity	0.66(−0.47, 1.80)	−0.12(− 0.90, 0.66)
Sleep problems		−0.60*(− 1.25, 0.041)	−1.14*(− 2.31, 0.018)
Edentulism		− 0.63**(− 1.23, − 0.038)	−1.27***(− 1.87, − 0.66)
Poor self-rated health		−1.52***(− 2.09,-0.95)	− 2.03***(− 2.50, − 1.56)
1 + ADL		−1.02***(− 1.47, − 0.56)	−2.77***(− 3.38, − 2.17)
Tobacco use		0.08(− 0.36, 0.52)	0.05(−0.44, 0.55)
Alcohol use		−0.99*(− 2.03, 0.035)	− 0.29(− 0.83, 0.25)
Physically inactive		−1.31***(− 1.78, − 0.84)	0.03(− 0.36, 0.43)
Self-reported depression		−1.35**(− 2.39, − 0.32)	−2.81*(−6.04, 0.42)
**Random part**			
Region		0.84(0.41, 1.73)	1.49(.74, 2.98)
PSU		1.96(1.70, 2.26)	2.56(2.09, 3.13)
Individual	–	7.44 (7.30, 7.58)	9.25(9.13, 9.37)
Observations	–	5787	10,934

^a^reference no formal education, ^b^reference never worked, ^c^reference lowest,^d^reference poorest,^e^reference male, ^f^reference urban, ^g^ reference currently married, ^h^ reference normal weight

*CI* confidence interval*** *p* < .001, ** *p* < .005, * *p* < .01

In India, father’s employment was positively associated with cognitive functioning. Further, height is strongly and positively associated with the cognitive functioning score. Older adults in the highest height quintile category in India (β = 1.81, CI: 1.15, 2.47, p < .001) and China (β = 2.23, CI: 1.60, 2.86, p < .001) had a better cognitive functioning score, respectively. Educational attainment of the study participants and wealth quintile were found as strong predictors of cognitive functioning. Age was negatively associated with cognitive functioning; this association was stronger for older adults in China. Furthermore, sleep problems and edentulism (loss of all natural teeth) were strongly associated with lower cognitive functioning. Being underweight was associated with lower cognitive functioning in India. Poor self-rated health was associated with lower cognitive functioning in India (β = − 1.52, CI: − 2.09,-0.95, *p* < .001) and China (β = − 2.03, CI: − 2.50,-1.56, p < .001). Similarly, the association between 1 + ADL limitations and cognitive functioning was significant in India (β = − 1.02, CI: − 1.47,-.56, p < .001) and China (β = − 2.77, CI: − 3.38,-2.17, p < .001). Both self-reported diagnosed depression and physical inactivity were significantly associated with cognitive functioning in India.

The association between life course SES and cognitive functioning is presented in Table 3. The combination of parental education and respondent’s education with cognitive functioning was positive when the mother’s education and respondents’ education was more than primary schooling in India and China. Similarly, the father’s education and respondent’s education showed a positive association with cognitive functioning in India and China. The education-based life course SES showed a positive association with cognitive functioning in India and China. Similarly, the employment-based life course SES showed positive association with cognitive functioning in India and China.

**Table 3:** Life course SES and cognitive functioning among older adults in India and China, WHO-SAGE Wave 1 (2007/10)

Life-course SES		India	China
		β (95% CI)	β (95% CI)
**Mother’s education**	**Own education**		
Less than primary	Less than primary	Ref	Ref
Greater than primary	Less than primary	0.74(−1.89, 3.38)	0.23(−2.66, 3.13)
Less than primary	Greater than primary	5.66***(5.14, 6.19)	4.39***(3.94, 4.83)
Greater than primary	Greater than primary	8.73***(7.60, 9.86)	6.91***(6.00, 7.81)
**Father’s education**	**Own education**	–	–
Less than primary	Less than primary	Ref	Ref
Greater than primary	Less than primary	0.88*(−0.08, 1.84)	1.15**(.027, 2.28)
Less than primary	Greater than primary	5.16***(4.58, 5.74)	4.23***(3.77, 4.69)
Greater than primary	Greater than primary	7.59***(6.85, 8.33)	6.45***(5.76, 7.14)
**Mother’s employment**	**Own employment**	–	–
Not employed	Not employed	Ref	Ref
Not employed	Employed	1.77***(0.62, 2.93)	1.10(−0.51, 2.73)
Employed	Not employed	1.14***(0.50, 1.78)	1.74***(0.92, 2.56)
Employed	Employed	0.70**(0.028, 1.38)	2.16***(1.30, 3.03)
**Father’s employment**	**Own employment**	–	–
Not employed	Not employed	Ref	Ref
Not employed	Employed	1.81*(−0.21, 3.84)	1.13*(− 0.18, 2.45)
Employed	Not employed	0.48(−1.87, 2.84)	1.79***(0.89, 2.68)
Employed	Employed	2.39**(0.39, 4.39)	2.26***(1.37, 3.15)
**Parental education**	**Own education**	–	–
Less than primary	Less than primary	Ref	Ref
Greater than primary	Less than primary	1.74(−1.83, 5.32)	3.62(−2.75, 10.0)
Less than primary	Greater than primary	5.04***(4.45, 5.63)	5.31***(4.54, 6.09)
Greater than primary	Greater than primary	9.75***(8.49, 11.0)	8.65***(6.25, 11.1)
**Parental employment**	**Own employment**	–	–
Not employed	Not employed	Ref	Ref
Not employed	Employed	3.99***(1.60, 6.38)	1.14(−0.58, 2.87)
Employed	Not employed	0.05(−2.41, 2.52)	1.86***(0.93, 2.80)
Employed	Employed	2.67**(0.48, 4.86)	2.46***(1.49, 3.43)

Separate regression analysis was performed for each predictor variable adjusting demographic and health variables

*CI* confidence interval *** p < .001, ** p < .005, * p < .01

**Fig. 2:**
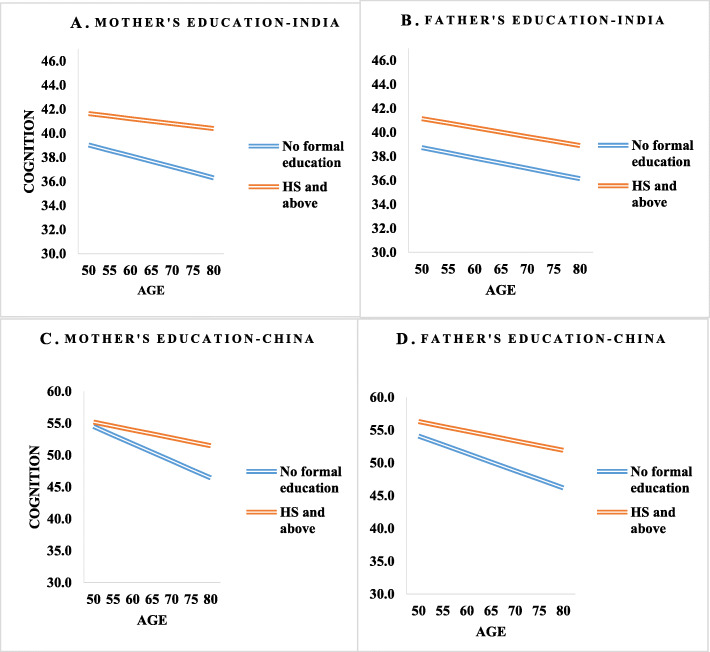
Predicted cognitive functioning score by parental education categories and age controlling socio-demographic, and health variables for India and China, WHO-SAGE Wave 1. **A** and **B**. Age pattern of cognition score of the study participants by mother’s and father’s education categories in India. **C** and **D**. Age pattern of cognition score of the study participants by mother’s and father’s education categories in China

Figure 2 shows the predicted cognitive functioning score for India and China by parental education across different ages. We used the interaction between age and parental education to show the change in the cognitive functioning score with age across parental education categories. Across different ages, cognitive functioning score was higher for older adults whose mother and father had high school and above education.

**Fig. 3:**
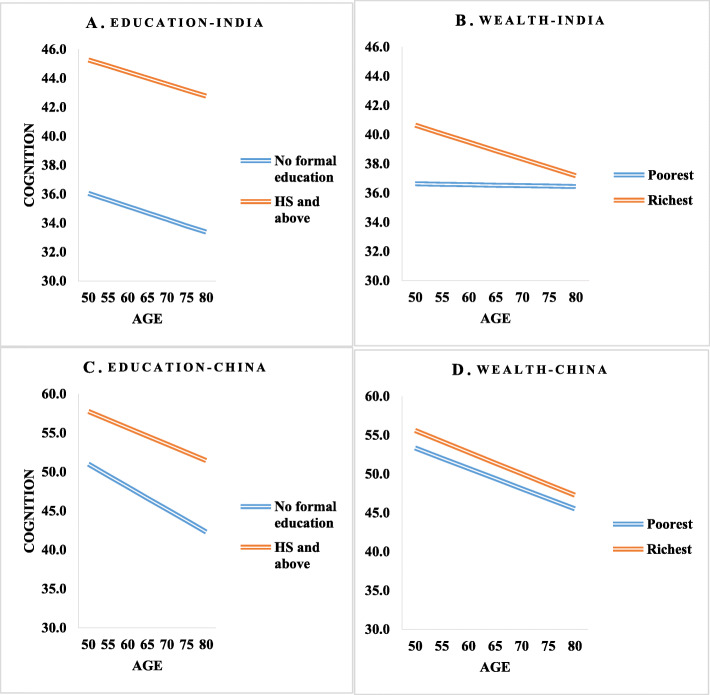
Predicted cognitive functioning score by education (study participants) and wealth quintile in relation to age controlling demographic, and health variables for India and China, WHO-SAGE Wave 1. **A** and **B**. Age pattern of cognition score by participant’s education and wealth quintile categories in India. **C** and **D**. Age pattern of cognition score by participant’s education and wealth quintile categories in China

Figure 3 shows that age pattern of cognitive functioning by respondent’s educational attainment and wealth quintile. In India and China, older adults who has completed higher secondary (HS) or above had higher cognitive functioning across different ages, more strongly in India. The cognitive functioning score is much lower across age among older adults who had no formal schooling than those who completed higher secondary (HS) or above in China. Wealthier older adults in India had higher cognitive functioning in 50–60 years of age, however, the wealth differences in cognitive functioning narrowed in older ages, suggesting the convergence of cognitive functioning by economic status at older ages.

## Discussion

In this study, we observed a significant and positive association between parental education, as a measure of childhood socioeconomic status and cognitive functioning of older adults in India and China. Furthermore, respondent’s socioeconomic status measured by educational attainment and household economic status (wealth quintile) were strongly associated with cognitive functioning. Particularly, the association of educational attainment and wealth quintile was stronger for India. The association between education and employment-based life course SES and cognition was significant, suggesting stable high socioeconomic status across the life course is important for higher cognitive functioning. The cognitive functioning score was lower across age among older adults whose parents had no schooling, particularly in China. Height showed a significant and positive association with cognitive functioning in India and China. However, the association was stronger for China. Cross-national and gender differences in cognitive functioning were notable. Older adults in India had lower cognitive functioning scores than their Chinese counterparts. Older women in India had lower cognitive functioning scores than men counterparts. Poor self-rated health, sleep problems and edentulism were negatively associated with cognitive functioning.

Overall, the association of childhood socioeconomic status and height with cognitive functioning was significant in India and China, suggesting the similarities in the association of childhood socioeconomic status and height in determining later life cognitive functioning. The results of this study on the association between parental education and later life cognitive functioning are consistent with the findings of previous studies conducted in China and South Africa suggesting the long-term effect of childhood circumstances in developing countries [27, 28, 31, 32]. We also observed lower cognitive functioning score among older adults whose parents had no schooling across different ages which highlighted the role of childhood circumstances; this is consistent with previous literature [50]. Previous studies including a recent study based on SHARE data for European countries found a significant positive relationship between childhood socioeconomic status and cognitive functioning in old age [24, 26, 28–30].

In this study, educational attainment was found as a strong predictor of cognitive functioning consistent with the findings of previous studies [51–53]. This association is consistent across different ages, suggesting the role of education in cognitive reserve. In China, the cognitive functioning score was higher among older adults with higher secondary (HS) and above education across different age as found in previous literature [50]. These results suggest the role of education in determining cognitive reserve in old age [54]. Low education is associated with a higher risk of cognitive impairment and dementia [55]. In this context, the role of education is seen as a protective factor of cognitive functioning across age. On the other hand, in India, wealthier older adults aged 50–60 had higher cognitive functioning, however, the wealth gradient narrowed after age 60 and above. The results of this study support the convergence of health hypothesis [56] in the Indian context [57]. In the health literature, health inequality tends to narrow in old age mainly as a result of mortality selection [56]. The results of the present study suggest a significant role of economic status as a protective factor of health status and access to nutrition among Indian older adults, specifically in the early age of 50–64.

In this study, height was significantly associated with higher cognitive functioning, as found in previous studies from high and middle-income countries [28, 40]. The association of height with health-related outcomes suggest a long-lasting relationship of childhood circumstances with later life outcomes. This association is stronger in low and middle-income settings which supports the hypothesis of a stronger effect of childhood circumstances in low-income settings [58, 59].

Overall, the findings of better cognitive functioning among older adults in China than India are likely to be result of better educational attainment and nutritional status among older adults in China. Poor self-rated health and 1 + ADL were negatively associated with cognitive functioning which suggested the significant role of general health and functional limitations on cognitive functioning in old age. Previous studies showed a similar negative association between poor self-rated health and cognitive functioning [60]. Poor self-rated health is a well-known indicator of general health which is a strong predictor of mortality [61]. The association between 1 + ADL and cognition was significant as shown in previous studies [62]. Oral health condition measured as a loss of all natural teeth was negatively associated with cognitive functioning [63].

The results of this study highlighted the significance of childhood socioeconomic status and height in determining late-life cognitive health in low and middle-income settings. Poor socioeconomic status across the life course continues to affect the individual outcomes on various health measures such as handgrip strength [64], frailty [65], and respiratory function [66]. Studies showed that childhood socioeconomic status played an important role in access to nutrition, health, and education which further affect the various individual outcomes throughout the life course [24, 34]. Especially, parental education has a significant role; educated parents are more likely to escape from the adverse environmental circumstances during pregnancy and better placed in providing better nurturing [67] and immunisation for their children [22, 23]. Childhood health and nutrition mediate as a human capital reserve and have a long-lasting impact on the health and wellbeing of their children. Also, children of educated parent’s escape from violence and multiple risk factors through better environmental circumstances with less violence. In contrast, children in poor childhood circumstances experience more health risk behaviour such as smoking and poor dietary habits [68]. Therefore, the role of better childhood circumstances is important in determining health and wellbeing across the life course.

### Strengths and limitations

This study has used measured height data to examine the relationship between height and cognitive functioning, while most of the previous studies used self-reported height [37, 40], in this study, we used two important childhood measures; height as a measure of childhood health and net-nutrition and childhood socioeconomic status. Previous studies focussed on either childhood socioeconomic status or height.

The limitations of the study are; the results of this study are based on cross-sectional data. Previous literature showed age-related decline in height [69, 70] which is not accounted for in the analysis. Jain and Ma (2020) [70] showed that height shrinkage is associated with lower cognition and health-related outcomes among the elderly. Furthermore, we relied on a few selected measures of childhood socioeconomic status, while several studies have used multiple measures of childhood socioeconomic status. Furthermore, the childhood socioeconomic status was assessed through the retrospective questions in the survey where there is a chance of misreporting. Moreover, in the SAGE survey, the measures of childhood health were not adequately collected unlike other surveys from high-income countries such as Health and Retirement Study (HRS) and Survey of Health, Ageing and Retirement in Europe (SHARE).

## Conclusions

The results of this study suggest that childhood socioeconomic circumstances and adult height as a proxy measure of childhood nutrition play an important role in determining later-life cognition independent of adult socioeconomic status, demographic and health risk factors. In India and China, parental education was significantly associated with cognitive functioning. Educational attainment and household economic status were significant factors in determining cognitive functioning among older adults in India and China. Results highlight the prominent role of parental education and own education across the life course in determining and maintaining better cognitive functioning. The role of wealth quintile in determining cognitive functioning is stronger in middle age to late middle age. Height is an important early life marker in determining cognitive functioning in later life suggesting the strong association of childhood nutrition and health. The findings of this study suggest improving health and nutrition in early childhood tend to have a long-lasting impact on cognitive functioning.

## Supplementary Information

**Additional file 1.**

## Data Availability

The data-sets used in the present study are available from the corresponding author on request.

## Abbreviations

SES: Socio-Economic Status
WHO-SAGE: World Health Organisation Study on global AGEing and adult health
HRS: Health and Retirement Study
SHARE: Survey of Health, Ageing and Retirement in Europe
SRH: Self-Rated Health
ADL: Activities of Daily Living
PSU: Primary **S**ampling Unit
