# Supplementary material for: Association of life course socioeconomic status and adult height with cognitive functioning of older adults in India and China
Source: BMC Geriatr. 2021 Jun 9;21:354. doi: 10.1186/s12877-021-02303-w (PMC8191062; doi:10.1186/s12877-021-02303-w)
Supplement: Supplementary file 1 — Additional file 1. [file 12877_2021_2303_MOESM1_ESM.docx]

***Cognitive functioning***

To understand the composite effect of cognition, we made a cognitive index combining four variables: verbal fluency, verbal recall, digit span forward and digit span backward.

Different cognition tests and the procedure used in the survey are;

**Verbal recall:** Interviewer read out a list of 10 commonly used words to the respondents and asked them to repeat again in some time.

**Digit span (forward and backward):**Participants were read a series of digits **(numbers)** and asked to immediately repeat them back. In the backward test, the person must repeat the numbers in reverse order. These tests measure concentration, attention, and immediate memory.

**Verbal fluency:** Participants were asked to produce as many animal names as possible in one-minute time span. This test assessed retrieval of information from semantic memory.

A composite cognitive score was derived using Principal Components Analysis (PCA), a mathematical tool which helps in creating a composite index using uncorrelated components, where each component captures the largest possible variation in the original variables. Selected raw scores for cognitive tasks were bundled into three domains (digit span, memory and executive functioning) to yield compound cognitive scores. This was done to condense the number of cognitive variables while refining the robustness of the underlying cognitive construct. We followed two steps to make a cognitive score:

*Step 1*: All four variables were in different scales. So first, we standardized these variables. A standardized variable (sometimes called a z-score or a standard score) is a variable that has been rescaled to have a mean of zero and a standard deviation of one. Each case's value on the standardized variable designates its difference from the mean of the primary variable in some standard deviations (of the original variable).

*Step 2*: PCA is a multivariate statistical technique used for extracting from a set of variables those few orthogonal linear combinations that capture the common information most successfully. Further, this index comprises both values, positive and negative. So we converted this index into a 0–100 scale which facilitates easier interpretation of the data. Higher scores indicate better cognitive functioning.

**Table 1: List of items used for calculation of household wealth, WHO-SAGE, 2007-10**

| **Question No.** | **Item details** |
| --- | --- |
| q0700 | Can you please tell me how many rooms there are in your home? |
| q0701 | How many chairs are there in your home? |
| q0702 | How many tables are there in your home? |
| q0703 | How many cars are there in your household? |
| q0704 | Does your home have electricity? |
| q0705 | Does anyone in your household have a bicycle? |
| q0706 | Does anyone in your household have a clock? |
| q0707 | Does anyone in your household have a bucket? |
| q0708 | Does anyone in your household have a washing machine for clothes? |
| q0709 | Does your household or anyone in your household have a dishwasher? |
| q0710 | Does anyone in your household have a refrigerator? |
| q0711 | Does anyone in your household have a fixed line telephone? |
| q0712 | Does anyone in your household have a mobile/cellular telephone? |
| q0713 | Does anyone in your household have a television? |
| q0714 | Does anyone in your household have a computer? |
| q0715 | Does anyone in your household have moped/scooter/motorcycle? |
| q0716 | Does anyone in your household have live-stock (cattle only)? |
| q0717 | Does anyone in your household have sewing machine? |
| q0718 | Does anyone in your household have radio/transistor/tape recorder? |

q0719 Does anyone in your household have bullock cart?

**Table 2 Distribution of Life course SES variables and height quintile for India and China, WHO-SAGE, 2007-10**

| **Life-course SES** | | India (%) | China (%) |
| --- | --- | --- | --- |
| **Mother’s education** | **Own education** |  |  |
| Less than primary | Less than primary | 61.01 | 40.48 |
| Greater than primary | Less than primary | 0.79 | 0.51 |
| Less than primary | Greater than primary | 34.5 | 52.87 |
| Greater than primary | Greater than primary | 3.7 | 6.14 |
| **Father’s education** | **Own education** |  |  |
| Less than primary | Less than primary | 56.64 | 38.43 |
| Greater than primary | Less than primary | 4.79 | 2.42 |
| Less than primary | Greater than primary | 23.22 | 43.01 |
| Greater than primary | Greater than primary | 15.36 | 16.13 |
| **Mother’s employment** | **Own employment** |  |  |
| Not employed | Not employed | 23.37 | 7.25 |
| Not employed | Employed | 3.63 | 1.37 |
| Employed | Not employed | 42.1 | 31.38 |
| Employed | Employed | 30.9 | 60 |
| **Father’s employment** | **Own employment** |  |  |
| Not employed | Not employed | 0.96 | 6.48 |
| Not employed | Employed | 25.99 | 2.1 |
| Employed | Not employed | 1.8 | 18.23 |
| Employed | Employed | 71.25 | 73.19 |
| **Parental education** | **Own education** |  |  |
| Less than primary | Less than primary | 67.69 | 44.01 |
| Greater than primary | Less than primary | 0.29 | 0.30 |
| Less than primary | Greater than primary | 27.7 | 49.07 |
| Greater than primary | Greater than primary | 4.32 | 6.62 |
| **Parental employment** | **Own employment** |  |  |
| Not employed | Not employed | 2.25 | 7.54 |
| Not employed | Employed | 9.49 | 1.52 |
| Employed | Not employed | 4.32 | 20.79 |
| Employed | Employed | 83.95 | 70.15 |
|  |  |  |  |
| **Height quintile distribution** | | | |
| Lowest |  | 19.2 | 20.4 |
| 2 |  | 20.4 | 20.1 |
| 3 |  | 19.7 | 22.5 |
| 4 |  | 20.0 | 19.7 |
| Highest |  | 20.7 | 17.2 |

**Table 3 Pearson Correlations of measures of life course socioeconomic status, height and demographic health risk factors and Cognitive Functioning, WHO-SAGE India, 2007-10**

| 1. Cognitive functioning | 1 | 2 | 3 | 4 | 5 | 6 | 7 | 8 | 9 | 10 | 11 | 12 | 13 | 14 | 15 | 16 | 17 | 18 |
| --- | --- | --- | --- | --- | --- | --- | --- | --- | --- | --- | --- | --- | --- | --- | --- | --- | --- | --- |
| 2. Mother’s education | 0.25* | 1 |  |  |  |  |  |  |  |  |  |  |  |  |  |  |  |  |
| 3.Father’s education | 0.31* | 0.50* | 1 |  |  |  |  |  |  |  |  |  |  |  |  |  |  |  |
| 4.Mother’s employment | -0.07* | -0.10* | -0.24* | 1 |  |  |  |  |  |  |  |  |  |  |  |  |  |  |
| 5.Father’s employment | 0.15* | 0.17* | 0.35* | 0.002 | 1 |  |  |  |  |  |  |  |  |  |  |  |  |  |
| 6.Height quintile | 0.35* | 0.35* | 0.04* | 0.03* | 0.03* | 1 |  |  |  |  |  |  |  |  |  |  |  |  |
| 7.Schooling | 0.55* | 0.35* | 0.50* | -0.19* | 0.22* | 0.10* | 1 |  |  |  |  |  |  |  |  |  |  |  |
| 8.Wealth Quintile | 0.33* | 0.24* | 0.33* | -0.10* | 0.17* | 0.16* | 0.39* | 1 |  |  |  |  |  |  |  |  |  |  |
| 9.Own employment | 0.26* | 0.03* | 0.02 | 0.17* | 0.17* | 0.05* | 0.29* | 0.05* | 1 |  |  |  |  |  |  |  |  |  |
| 10.Age | -0.17* | -0.05* | -0.07* | -0.02 | -0.03* | -0.16* | -0.11* | -0.01 | 0 | 1 |  |  |  |  |  |  |  |  |
| 11.Sex | -0.30* | 0.01 | 0.01 | 0.02* | 0.009 | -0.001 | -0.37* | -0.003 | -0.53* | -0.06* | 1 |  |  |  |  |  |  |  |
| 12.Residence | -0.20* | -0.27* | -0.30* | 0.12* | -0.17* | -0.03* | -0.27* | -0.31* | -0.03* | 0.002 | -0.03* | 1 |  |  |  |  |  |  |
| 13.Marital status | -0.23* | -0.04* | -0.05* | -0.001 | -0.02 | -0.09* | -0.21* | -0.08* | -0.17* | 0.28* | 0.31* | -0.01 | 1 |  |  |  |  |  |
| 14.Body mass index | 0.20* | 0.15* | 0.20* | -0.07* | 0.09* | 0.06* | 0.20* | 0.30* | -0.01 | -0.11* | 0.07* | -0.21* | -0.05* | 1 |  |  |  |  |
| 15.Sleep problems | -0.16* | -0.03* | -0.02* | 0 | -0.05* | -0.051* | -0.1200* | -0.1095* | -0.104* | 0.121* | 0.09* | 0.058* | 0.095* | -0.059* | 1 |  |  |  |
| 16.Edentulism | -0.08* | -0.036* | -0.038* | -0.014 | -0.022 | -0.071* | -0.0424* | 0.0053 | -0.04* | 0.239* | 0.02 | -0.007 | 0.099* | -0.051* | 0.087* | 1 |  |  |
| 17.Poor self-rated health | -0.22* | -0.05* | -0.02* | -0.04* | -0.0252* | -0.083* | -0.1227* | -0.1318* | -0.09* | 0.187* | 0.051* | 0.07* | 0.113* | -0.120* | 0.317* | 0.06* | 1 |  |
| 18.Self-reported depression | -0.06* | -0.03* | -0.03* | -0.04* | -0.1571* | -0.0067 | -0.0182 | -0.0064 | -0.06* | 0.008 | -0.035* | 0.029* | -0.008 | -0.007 | 0.066* | 0.04* | 0.08* | 1 |

*Significant at p < .005,

**Table 4 Pearson Correlations of measures of life course socioeconomic status, height and demographic health risk factors and Cognitive Functioning, WHO-SAGE China, 2007-10**

| 1.       Cognitive functioning | 1 | 2 | 3 | 4 | 5 | 6 | 7 | 8 | 9 | 10 | 11 | 12 | 13 | 14 | 15 | 16 | 17 | 18 |
| --- | --- | --- | --- | --- | --- | --- | --- | --- | --- | --- | --- | --- | --- | --- | --- | --- | --- | --- |
| 2. Mother’s education | 0.244* | 1 |  |  |  |  |  |  |  |  |  |  |  |  |  |  |  |  |
| 3.Father’s education | 0.295* | 0.564* | 1 |  |  |  |  |  |  |  |  |  |  |  |  |  |  |  |
| 4.Mother’s employment | 0.072* | 0.260* | 0.132* | 1 |  |  |  |  |  |  |  |  |  |  |  |  |  |  |
| 5.Father’s employment | 0.175* | 0.271* | 0.427* | 0.588* | 1 |  |  |  |  |  |  |  |  |  |  |  |  |  |
| 6.Height quintile | 0.266* | 0.180* | 0.185* | -0.035* | 0.076* | 1 |  |  |  |  |  |  |  |  |  |  |  |  |
| 7.Schooling | 0.469* | 0.311* | 0.379* | -0.001 | 0.191* | 0.325* | 1 |  |  |  |  |  |  |  |  |  |  |  |
| 8.Wealth Quintile | 0.349* | 0.2166* | 0.248* | 0.033* | 0.116* | 0.231* | 0.3920* | 1 |  |  |  |  |  |  |  |  |  |  |
| 9.Own employment | 0.249* | 0.1972* | 0.2555* | 0.1629* | 0.325* | 0.143* | 0.3445* | 0.2910* | 1 |  |  |  |  |  |  |  |  |  |
| 10.Age | -0.339* | -0.1441* | -0.1574* | -0.1810* | -0.193* | -0.244* | -0.2848* | -0.1620* | 0.0091 | 1 |  |  |  |  |  |  |  |  |
| 11.Sex | -0.124* | -0.0016 | -0.0121 | 0.0037 | 0.0012 | -0.0081 | -0.2169* | -0.0077 | -0.1176* | -0.0058 | 1 |  |  |  |  |  |  |  |
| 12.Residence | -0.227* | -0.2711* | -0.3388* | 0.1100* | -0.1544* | -0.2612* | -0.4148* | -0.3076* | -0.4791* | -0.0889* | -0.0513* | 1 |  |  |  |  |  |  |
| 13.Marital status | -0.2023* | -0.0468* | -0.0655* | -0.0223* | -0.0540* | -0.1342* | -0.2343* | -0.1733* | -0.0550* | 0.3325* | 0.1566* | -0.0095 | 1 |  |  |  |  |  |
| 14.Body mass index | 0.0549* | 0.0138 | -0.0035 | -0.0399* | -0.0077 | 0.0511* | 0.0627* | 0.1154* | 0.0296* | -0.0563* | 0.0869* | -0.1115* | -0.0494* | 1 |  |  |  |  |
| 15.Sleep problems | -0.0869* | -0.0084 | -0.0151 | -0.0031 | -0.0137 | -0.0255* | -0.0562* | -0.0438* | -0.0385* | 0.0586* | 0.0501* | 0.0174* | 0.0388* | -0.012 | 1 |  |  |  |
| 16.Edentulism | -0.1983* | -0.0804* | -0.0987* | -0.0737* | -0.1078* | -0.1054* | -0.1694* | -0.1253* | -0.1103* | 0.3040* | 0.0230* | 0.0649* | 0.1367* | -0.038* | 0.05* | 1 |  |  |
| 17.Poor self-rated health | -0.2219* | -0.0768* | -0.0929* | 0.0371* | -0.0038 | -0.1109* | 0.1752* | -0.1743* | -0.0993* | 0.1342* | 0.0589* | 0.1073* | 0.0917* | -0.017* | 0.17* | 0.114* | 1 |  |
| 18.Self-reported depression | -0.0122 | -0.0103 | -0.0065 | -0.0113 | 0.0131 | 0.0122 | 0.0036 | -0.0062 | 0.0146 | -0.0093 | 0.0134 | -0.0262* | 0.0013 | 0.002 | 0.01* | -0.001 | 0.0442* | 1 |

*Significant at p < .005,
